# Brood-partitioning behaviour in unpredictable environments: hedging the bets?

**DOI:** 10.1007/s00265-015-1913-1

**Published:** 2015-04-10

**Authors:** Magdalena Erich, Max Ringler, Walter Hödl, Eva Ringler

**Affiliations:** Department of Integrative Zoology, University of Vienna, Althanstrasse 14, 1090 Vienna, Austria; Messerli Research Institute, University of Veterinary Medicine Vienna, Veterinärplatz 1, 1210 Vienna, Austria

**Keywords:** Brood spreading, Bet-hedging, Parental care, Tadpole transport, Amphibians

## Abstract

Spreading reproduction across time or space can optimize fitness by minimizing the risks for offspring survival in varying and unpredictable environments. Poison frogs (Dendrobatidae) are characterized by complex spatial and reproductive behaviour, such as territoriality, prolonged courtship and parental care. The partitioning of larvae from terrestrial clutches across several water bodies is mainly known from species with carnivorous tadpoles that allocate their tadpoles in very small pools, where limited food availability is accompanied by an increased risk of cannibalism. However, little is known about the deposition behaviour of non-carnivorous species that use medium-sized to large pools. In the present study, we investigated whether the Neotropical poison frog *Allobates femoralis* exhibits brood-partitioning behaviour when males transport tadpoles 3 weeks after oviposition. We sampled 30 artificial water bodies for tadpoles, which we genotyped at seven highly polymorphic microsatellite loci. Based on the reconstructed pedigree, we show that *A. femoralis* males distribute larvae of single and of successive clutches across several water bodies. The number of pools used was significantly associated with the number of clutches per male. Ninety-three percent of the males that were assigned to more than one clutch spread their tadpoles across several water bodies. Given the highly variable and unpredictable biotic and abiotic conditions in tropical rainforest, at the spatial scale of the study species’ behaviour, we interpret this behaviour as bet-hedging to improve offspring survival.

## Introduction

Variable and unpredictable environmental conditions are expected to favour the evolution of bet-hedging strategies (Beaumont et al. 2009; Simons 2011). By spreading the risk of mortality across time and/or space, individuals can optimize fitness by reducing the variance in reproductive success in favour of long-term risk reduction (Hopper 1999; Crean and Marshall 2009; Olofsson et al. 2009; Simons 2011). Several studies have demonstrated fitness benefits of diversified reproductive strategies; e.g. allocating reproduction across several mating partners (Fox and Rauter 2003; Mäkinen et al. 2007; Sarhan and Kokko 2007; Garcia-Gonzalez et al. 2015), distributing eggs across several sites (Root and Kareiva 1984; Byrne et al. 2009; Andersson and Åhlund 2012) and variably initiating egg incubation in order to promote hatching asynchrony (Laaksonen 2004). However, compared to the comprehensive theoretical literature on bet-hedging strategies, empirical datasets from natural populations, particularly on parental behaviours *after* hatching, are rather scarce.

Among anurans, parental care is mainly found in terrestrial breeders in the humid tropics (Wells 2007). Terrestrial oviposition has evolved independently several times, presumably as an adaptation to high aquatic predation pressure on eggs (Magnusson and Hero 1991) and the risk of reduced fertilization success due to stray sperm from rivals in aquatic environments (Roberts and Byrne 2011). The tadpoles of most terrestrial breeders still complete their development until metamorphosis in water, a dilemma which some species solve by transporting their tadpoles to aquatic sites (Wells 2007).

Pre-metamorphic mortality in amphibians is around 90 % in most species (Vonesh and De la Cruz 2002), caused by abiotic and biotic factors, such as predation (Magnusson and Hero 1991), pond desiccation (Richter-Boix et al. 2011), inter- and intra-specific competition for food (Gonzalez et al. 2011) as well as parasitism and pathogen infections (Kriger and Hero 2007; Rhoden and Bolek 2011). As the choice of high-quality larval developmental sites has profound effects on offspring survival and thus on the parent’s reproductive success, selection should therefore drive the evolution of parental behaviours that assess the quality of breeding sites in order to maximize offspring growth and survival. Such discriminatory behaviour regarding larval deposition sites has been shown in several anuran species (Spieler and Linsenmair 1997; Summers 1999; Summers and McKeon 2004; Schulte et al. 2011). However, natural environments of amphibian larvae are also characterized by highly unpredictable variability (e.g. weather, predators, etc.), making it almost impossible for the transporting parent to reliably predict the future quality of a pool for the entire period of larval development. Depositing offspring (of single and/or successive clutches) in several pools therefore would constitute a suitable bet-hedging strategy to minimize the risk of total offspring loss in face of environmental uncertainty (cf. Byrne et al. 2009).

Neotropical poison frogs (Dendrobatidae) show complex behaviours such as territoriality, elaborate courtship and parental care. All species deposit their eggs outside of water, and after hatching, most of them transport the tadpoles to water bodies, where they complete their larval development until metamorphosis (Lötters et al. 2007). In some dendrobatids, adults select deposition sites according to food availability (Poelman et al. 2013) or the presence of predators or conspecifics (Brown et al. 2008; Schulte et al. 2011; McKeon and Summers 2013; Rojas 2014). Species that have carnivorous tadpoles which they transport to small pools, such as bromeliads or small tree holes, are known to distribute their larvae across several sites (Caldwell and de Araújo 1998; Summers 1999). Limited food availability accompanied by an increased risk of cannibalism presumably selected for this behaviour (Summers and Amos 1997; Brown et al. 2008; see also Stynoski et al. 2014). Little is known about the deposition behaviour of non-carnivorous species that use medium-sized pools, where nutrients are usually not limited.

Given the highly unpredictable biotic and abiotic impacts in temporal aquatic environments, we expect that brood-partitioning has evolved also in species that use medium-sized pools. We tested this hypothesis in the Neotropical poison frog *Allobates femoralis* by using field observations and microsatellite genotypes of adults and tadpoles from one reproductive season in order to identify whether males distribute their tadpoles from successive and/or single clutches across several water bodies.

## Methods

### Study species

Males of the Neotropical frog *A. femoralis* (Boulenger 1884; Anura: Dendrobatidae) are highly territorial and announce territory possession by a prominent advertisement call (Narins et al. 2003; Ringler et al. 2011). Females are inter-dispersed between male territories and show non-aggressive site fidelity (Ringler et al. 2009, 2012). Pair formation, courtship and mating occur in male territories (Roithmair 1992; Montanarin et al. 2011). Both sexes are highly polygynandrous throughout the reproductive season. Females can produce one clutch every 8 days on average (Weygoldt 1980, in captivity) and males were observed to guard up to five clutches simultaneously (Ursprung et al. 2011). Tadpole transport by male *A. femoralis* takes place 15–20 days after oviposition, which carry on average 8 (range = 1–25) tadpoles to small to medium-sized terrestrial pools (e.g. medium-sized temporal pools, floodplains, peccary wallows, footprints, palm fronds, holes in fallen trees), usually located outside a male’s territory (Ringler et al. 2013). In captivity, *A. femoralis* distribute their tadpoles when provided with several water pools, and tadpoles require 40–50 days of aquatic development until metamorphosis (Weygoldt 1980).

Our study population of *A. femoralis* is located in a lowland rainforest near the field camp ‘Saut Pararè’ (4° 02′ N, 52° 41′ W) in the nature reserve ‘Les Nourages’, French Guiana (details in Ursprung et al. 2011; Ringler et al. 2014). Thirty artificial pools (30 × 30 × 20 cm) were placed in a 6 by 5 array with ∼10 m distance between pools in the centre of our study plot in 2009, originally for another study on the effects of reproductive resource supplementation in *A. femoralis* (Ringler et al. 2015). Each pool was filled with approximately 500 cm^3^ of leaf litter to match natural forest floor cover and filled with rainwater.

### Sampling and genotyping

Data on the spatial locations of all adult individuals as well as microsatellite genotypes were already available from a previous study (Ringler et al. 2013). We sampled the artificial pools in January and April 2010 to capture tadpole-transport events over the entire study period and to avoid sampling larvae from one clutch repeatedly. We randomly sampled approximately one third of all tadpoles per pool and sacrificed them in 96 % ethanol. Additionally, we assessed the presence of potential predators of *A. femoralis* at the first sampling event (i.e. we noted all potential predators of *A. femoralis* tadpoles that were present at the respective pool). DNA extraction, genotyping and parentage assignments followed the protocol of Ursprung et al. (2011). We used all markers that were available for the Guiana population when samples were genetically analysed.

### Brood-partitioning behaviour

We reconstructed patterns of tadpole deposition by *A. femoralis* males via parentage assignments of larval and adult genotypes. Specifically, we aimed to reveal whether males distribute their larvae of single and/or successive clutches across multiple pools. We only included tadpoles with at least five unambiguously genotyped loci. Given that females produce multiple clutches throughout the breeding season, but rarely mate with the same male twice (Ursprung et al. 2011; Ringler et al. 2012), and males only transport tadpoles from a single clutch at a time (Ringler et al. 2013), we were able to distinguish between inter- and intra-clutch partitioning of *A. femoralis* males. We assumed that tadpole full-siblings (as inferred by the parentage analysis) from the same sampling cohort (January or April) belonged to the same clutch. Full-siblings that were sampled in different cohorts, as well as paternal half-sibs, were considered to originate from separate clutch depositions and were thus treated as distinct transportation events of the respective fathers. Given the larval development time in water until metamorphosis of about 45 days, tadpoles from the first sampling cohort would have already left the pool at the second sampling event. We only included clutches that were represented by at least two full-sibs, and for the analyses of inter-clutch partitioning, we considered only males that were assigned to at least two clutches. All statistical tests were performed in IBM SPSS Statistics 20.0.0. Normality of variables was tested with the Kolmogorov–Smirnov test. Medium values, interquartile ranges (iqr) and non-parametric tests were applied in cases where normality was rejected.

## Results

**Fig. 1:**
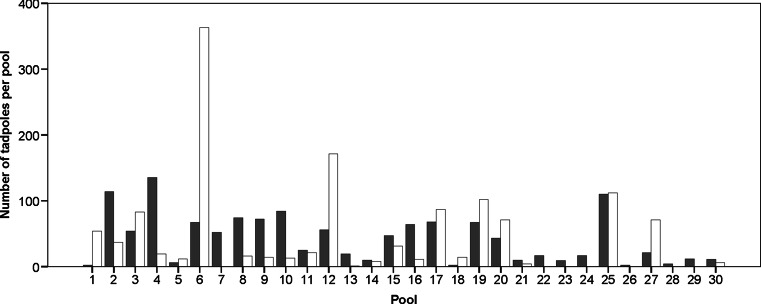
Total number of tadpoles detected in individual pools at both samplings. *Grey bars* refer to the first and *white bars* to the second sampling, respectively

**Fig. 2:**
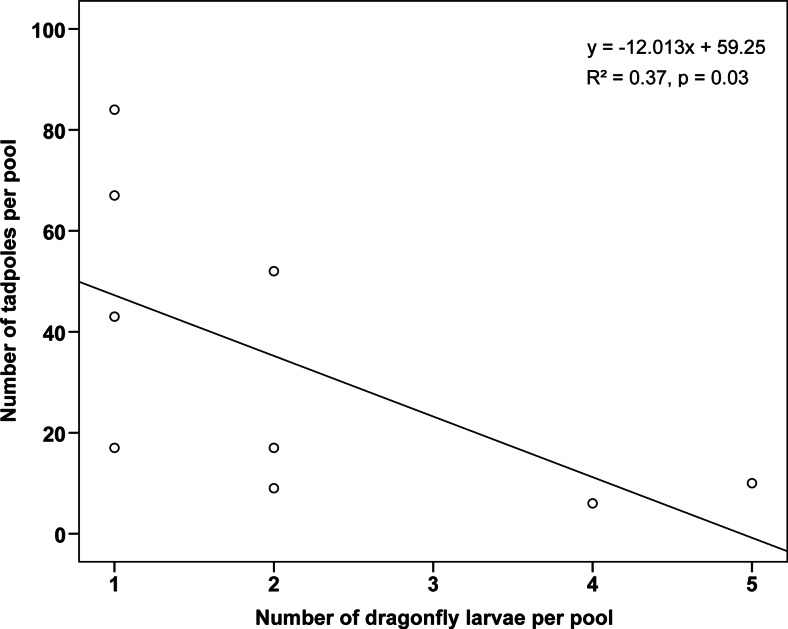
Total number of tadpoles in relation to the number of dragonfly larvae per pool

We counted a total of 2595 *A. femoralis* tadpoles in our artificial pools across both samplings, with pools containing a median number of 19 tadpoles per pool (iqr = 7.5–67.25, max = 363, Fig. 1). Dragonfly larvae were the most common aquatic predators in our artificial pools. Pools contained also a few singular carnivorous tadpoles of *Dendrobates tinctorius*, which usually get deposited in elevated tree holes. We observed that the number of tadpoles per pool significantly decreased with increasing number of dragonfly larvae (*n* = 9, Spearman’s *ρ* = −0.717, *y* = −12.013*x* + 59.25, *R*^2^ = 0.37, *p* = 0.03; Fig. 2). Pools without any dragonfly larvae contained up to 135 tadpoles (mean ± SD = 46.14 ± 39.38). During the study period, we sporadically observed the presence of other potential predators of *A. femoralis* tadpoles in and at the pools: spiders, snakes (e.g. *Helicops angulatus*), terrestrial crabs and twist-necked turtles (e.g. *Platemys platycephala*). In three cases, we observed a dramatic decrease in water quality caused by fallen fruits, dead animals and faeces (e.g. of howler monkeys) that started to ferment and rot inside the pools, which lead to a total loss of tadpoles inside the affected pools. We sampled in total 414 tadpoles by collecting up to 19 tadpoles per pool and sampling session (median = 6, iqr = 3.75–10).

**Fig. 3:**
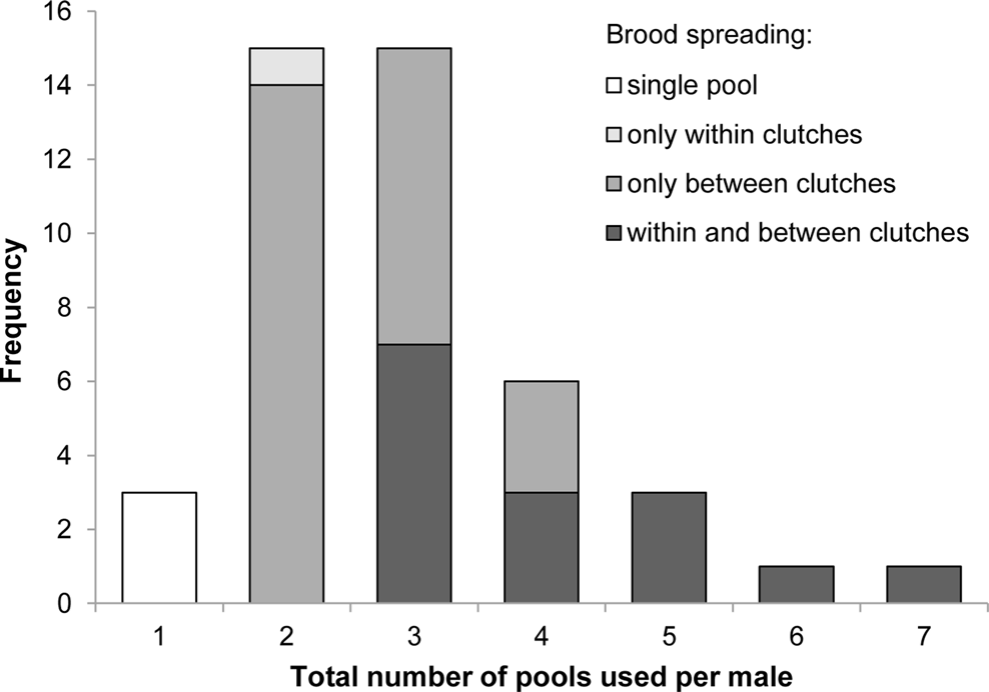
Histogram of pool use for larval deposition by *A. femoralis* males. Only males with at least two clutches are considered in this graph

**Fig. 4:**
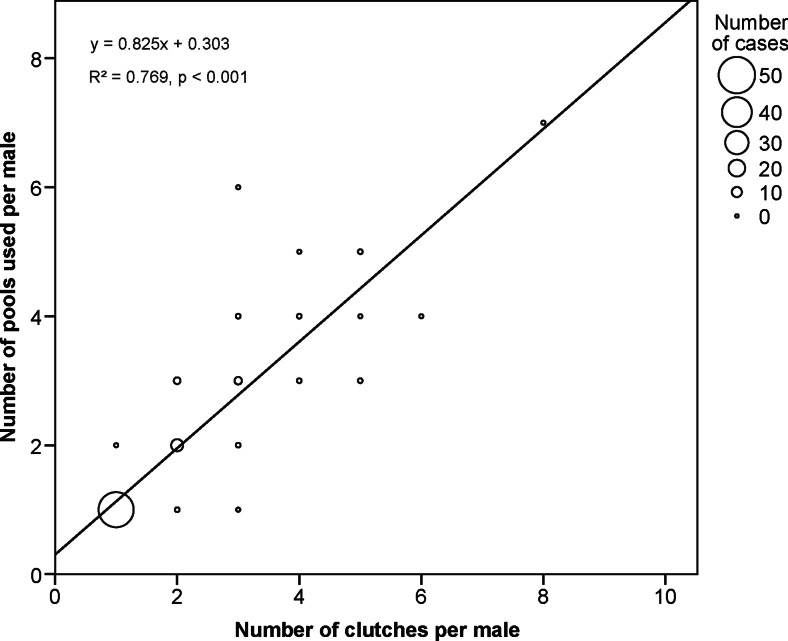
Relation between the number of clutches per male and the number of pools used per male. *Dot size* indicates the number of cases

We had to exclude 19 tadpoles from further analyses due to low PCR amplification success. Of the remaining 395 tadpoles, 340 could be unambiguously assigned to a known father from the 2010 population. Ninety-three (43.9 %) of the 212 *A. femoralis* males in our study had produced offspring. Our genetic analysis indicated that of these 93 males, 65 sired more than 2 of the analysed tadpoles and 44 males sired tadpoles from more than one clutch. Forty-one (93.18 %) of the 44 males distributed their tadpoles (from single or successive clutches) across several water bodies (Fig. 3). Seventeen males spread the larvae of single clutches over either two (*n* = 14) or three (*n* = 3) pools, respectively. Not a single male with more than three clutches carried its tadpoles to only one single pool (Fig. 4). On average, we found larvae of three males within one pool at a given sampling session (iqr = 1.75–4). Individual males who were assigned to more than one tadpole used a median of two pools (iqr = 1–3, *n* = 65). The number of pools used per male was significantly associated with the number of identified clutches (*n* = 93, *r* = 0.877, linear regression fit: *y* = 0.825*x* + 0.303, *R*^2^ = 0.769, *p* < 0.001; Fig. 4). One additional clutch per male translated into, on average, 0.825 (standard error = 0.047) additional used pools. This relationship also remained significant when all males with only one assigned tadpole were removed from the analysis, but the effect size was then reduced to additional 0.44 (standard error = 0.064) pools, on average, per added clutch (*n* = 65, *r* = 0.843, linear regression fit: *y* = 0.789*x* + 0.444, *R*^2^ = 0.710, *p* < 0.001).

## Discussion

In this study, we provide evidence for brood-partitioning behaviour in *A. femoralis*. The number of pools where males used to deposit their tadpoles increased significantly with their number of clutches. On average, 0.82 additional pools were used per additional clutch by each male. All males with more than three clutches used multiple pools for larval deposition. This demonstrates that *A. femoralis* males generally distribute their offspring from successive clutches, and to a lesser extent also from single clutches, across several pools.

Based on the heterogeneity of ephemeral aquatic environments, there are presumably large differences in quality with respect to tadpole development. Small natural water bodies at our study site were observed to quickly dry out when exposed to direct sunlight in periods of little rainfall (cf. Roth and Jackson 1987; Summers 1999; pers. obs. by all authors). Large pools are less susceptible to desiccation, but in turn are associated with increased predation risk by invertebrate larvae (Alford 1999). In our experimental design, pool size was constant (approx. 20 L), because pools were originally established for a study on reproductive resource supplementation (Ringler et al. 2015). We found dragonfly larvae and to a lesser extent heterospecific carnivorous tadpoles (e.g. *D. tinctorius*) as common aquatic predators of *A. femoralis* tadpoles at our study site. Also, spiders, snakes and tortoises were identified as potential predators of *A. femoralis* tadpoles. Although cannibalism is common in many dendrobatids (Caldwell and de Araújo 1998; Summers and Symula 2001), *A. femoralis* tadpoles do not show this behaviour (Weygoldt 1980, obs. in captivity; ER pers. obs.). Consequently, male *A. femoralis* do not avoid placing their offspring with other conspecific tadpoles.

In pools where dragonfly larvae were present, the number of tadpoles per pool significantly decreased with increasing number of dragonfly larvae. We cannot unambiguously distinguish whether this effect was caused by prior predation by the dragonfly larvae or whether it is a result of differential deposition of fewer tadpoles into pools with many dragonfly larvae. It has been shown by McKeon and Summers (2013) that *A. femoralis* males try to assess the presence of predators at specific water bodies immediately *before* larval deposition and adjust their deposition behaviour accordingly. However, predators could also enter the pool *after* tadpoles have been released. Thus, even if transporting males assess predator presence prior to tadpole deposition, these males cannot be certain of a low predation risk for their brood in the near future. In addition to predation, other confounding and highly unpredictable factors were identified, such as fruits, faeces or dead animals that fell into the pools and severely degraded water quality (i.e. no surviving tadpoles were detected inside these pools). In contrast, other pools contained up to 363 tadpoles when they were sampled (Fig. 1). We conclude that there are highly diverse and unforeseeable threats regarding tadpole development across pools—a crucial prerequisite for bet-hedging strategies to evolve.

The costs of tadpole transport for the carriers, such as conspicuousness to predators (Crump 1995), energetic effort (Simon 1983), or lost mating opportunities (Townsend 1986) will increase with the time spent distributing tadpoles. We expect increased costs for both inter- and intra-clutch partitioning (e.g. increased searching effort or longer duration when allocating tadpoles of a single clutch across multiple pools) compared to males always using the same pool for larval deposition. However, the resulting increase in tadpole survival might outweigh the associated costs for the transporting individual. Our data do now allow the assessment of whether patterns of tadpole distribution ultimately influence individual reproductive success in *A. femoralis*. But given the high variation in quality among (natural) water bodies, the low predictability of risks for the entire period of larval development and the presumably small increase in costs for the transporting parent (compared to single-pool use), a strong selective benefit of bet-hedging strategies can be assumed (cf. Leimar 2009). The allocation of larvae across multiple sites may act as a risk-spreading strategy to overcome the risk of total offspring loss, by combining advantages from between- and within-generation bet-hedging (i.e. inter- and intra-clutch partitioning), respectively (cf. conservative bet hedging: Philippi and Seger 1989; see also Summers 1990 and Starrfelt and Kokko 2012). We hypothesize that such brood-spreading behaviour has evolved as an adaptation to environmental uncertainty.

We cannot unambiguously differentiate whether males actively approached specific pools or whether they roamed through the area in search of suitable water bodies. However, *A. femoralis* males can accurately navigate in familiar areas around their territories (Pašukonis et al. 2014a, b), and previous studies indicate a strategic behaviour rather than an aimless search for suitable tadpole deposition sites (Ringler et al. 2013). As naturally occurring tadpole-deposition sites are generally neither common nor evenly distributed, the use of known pools would be preferable to repeated random searching. Preliminary data on tadpole transport trajectories show straight approach trajectories (Pašukonis, unpublished data). Hence, we presume that males actively approach the pools they know to distribute their larvae.

The observed brood-partitioning behaviour might also have causes other than bet-hedging, such as reduced competition among kin or resource depletion (cf. Smith 1990; Saidapur and Girish 2001; see also Pfennig 1990). Given that *A. femoralis* tadpoles are herbivorous and nutrients in medium-sized pools are not a limiting factor (Brown et al. 2008 and references therein), we expect that the negative impact of tadpole density is rather negligible. The variation in the number of tadpoles across pools was high (Fig. 1); thus, it is highly unlikely that the observed brood-spreading strategy has evolved as a mechanism to avoid high larval density in water bodies. However, the observed brood-partitioning behaviour is not necessarily driven by one single mechanism. Beside the benefits of bet-hedging, other positive effects due to allocating kin across space might have helped to shift the cost-benefit ratio in favour of a brood-spreading strategy.

In dendrobatid frogs, we find the evolutionary transition from species carrying many tadpoles (e.g. whole clutches) into large water bodies to species that allocate single tadpoles each to distinct small-sized pools, such as bromeliad axils or other phytotelmata (Weygoldt 1987; Brown et al. 2010). We hypothesize that the group-wise partitioning of tadpoles, particularly of single clutches, in species that use medium-sized pools might have been a transitional evolutionary step towards the deposition of only single tadpoles in very small pools (cf. Summers and McKeon 2004). While large bodies of water are generally associated with high predation risk, small pools offer almost predator-free environments (see Brown et al. 2008 and references therein). At the same time, food availability for larvae decreases with decreasing size of the water body. Consequentially, species with carnivorous tadpoles allocate their larvae singly into small phytotelmata to avoid larval cannibalism among siblings (Summers 1999; Summers and McKeon 2004) and regularly return to the deposition site in order to provision their young with unfertilized eggs (Poelman and Dicke 2007; Brown et al. 2009). However, these modalities are only the two extremes of a continuum, with many dendrobatid species actually using medium-sized pools for larval deposition (Brown et al. 2010). In our study, we show that brood-partitioning behaviour can occur in a species that uses medium-sized water bodies. This finding demonstrates that brood-spreading behaviour can evolve also in the absence of cannibalism and fully independent from post-deposition provisioning of aquatic larvae.
